# Peer teacher training (PTT) program for health professional students: interprofessional and flipped learning

**DOI:** 10.1186/s12909-017-1037-6

**Published:** 2017-12-04

**Authors:** Annette Burgess, Chris Roberts, Christie van Diggele, Craig Mellis

**Affiliations:** 1 Education Office, Sydney Medical School, The University of Sydney, Edward Ford Building A27, Sydney, NSW 2006 Australia; 2 Sydney Medical School – Central, The University of Sydney, Sydney, NSW 2006 Australia

**Keywords:** Healthcare education, Interprofessional, Peer teacher training

## Abstract

**Background:**

The need for developing healthcare professional students’ peer teaching skills is widely acknowledged, and a number of discipline-based peer teacher training programs have been previously reported. However, a consensus on what a student peer teaching skills program across the health professions should entail, and the associated benefits and challenges, has not been previously described. The purpose of this study was to demonstrate the design and implementation of an interprofessional Peer Teacher Training (PTT) program, and explore outcomes and participant perceptions, using Experience-Based Learning (ExBL) theory.

**Methods:**

In 2016, an interprofessional team of academics from across three healthcare faculties: Medicine, Pharmacy and Health Sciences, developed and implemented a six module, flipped learning, interprofessional PTT program. Pre- and post questionnaires, using a Likert scale of 1–5, as well as open ended questions, were distributed to students. Descriptive statistics were used to analyse quantitative data, and thematic analysis was used to analyse qualitative data.

**Results:**

Ninety senior students from across the three faculties participated. Eighty nine percent of participants completed a pre- and post-course questionnaire. Students felt the required pre-class preparation, including online pre-reading, discussion board, videos, and teaching activities enhanced their face-to-face learning experience. In class, students valued the small-group activities, and the opportunities to practice their teaching skills with provision of feedback. Students reported increased confidence to plan and deliver peer teaching activities, and an increased awareness of the roles and responsibilities of health professionals outside of their own discipline, and use of different terminology and communication methods. Students’ suggestions for improving the PTT, included; less large group teaching; more online delivery of theory; and inclusion of a wider range of health professional disciplines.

**Conclusion:**

The PTT program provided a theoretically informed framework where students could develop and practice their teaching skills, helping to shape students’ professional values as they assume peer teaching responsibilities and move towards healthcare practice. The flipped learning, interprofessional format was successful in developing students’ skills, competence and confidence in teaching, assessment, communication and feedback. Importantly, participation increased students’ awareness and understanding of the various roles of health professionals.

## Background

The importance of developing teaching skills within the health professions is widely acknowledged, and a number of teacher training programs within disciplines of university healthcare education curricula have been previously described [1, 2]. There are four key reasons identified for developing the teaching skills of health professional students [2, 3]. These include 1) preparation for participation in peer teaching activities at university; 2) preparation for future roles as healthcare teachers in the workforce; 3) to assist in the development of communication skills, which may improve interaction with patients; and 4) to develop a better understanding of teaching strategies, which may in turn help students become better learners [1–3]. However, a consensus on what a student peer teaching skills program across the health professions should entail, and the associated benefits and challenges, has not been previously described or investigated. An opportunity to investigate these issues arose when we designed, implemented and evaluated an innovative peer teacher training program at the University of Sydney, that adopted an interprofessional and flipped classroom approach. The overall aims of the program were to: promote student engagement in the development of learning and teaching, assessment and feedback skills; and promote engagement with interprofessional education.

### Interprofessional education in the peer teacher training context

Recent literature indicates that interprofessional education during healthcare training leads to improvements in leadership, collaboration and communication between healthcare teams, ultimately improving patient safety [4–6]. Although this link provides a powerful reason to implement interprofessional learning activities within university healthcare curricula, there are limited examples available [7, 8]. In fact, healthcare curricula have been described as “out-dated” and “static”, meaning graduates are “ill equipped” to work within increasingly complex healthcare systems [8]. Barriers to the implementation of interprofessional curricula within healthcare education include negative attitudes, with healthcare professionals preferring the silos of their individual disciplines; and pragmatic issues, such as the logistics of timetabling across disciplines.

It has been suggested that engagement in interprofessional learning activities within the senior years of university healthcare education, at a point in time when students have a well developed understanding of their own professional responsibilities, may assist in development of their professional identity [9]. Further to this, it is expected that by their senior years, students will have sound clinical knowledge and skills to draw on when teaching their junior peers.

### The advantages of a flipped classroom approach

The flipped classroom approach promotes efficiency in learning, and the construction of knowledge through social interaction. It offers a pedagogical method where learners prepare for teaching by making themselves familiar with the material prior to class [10]. The advantages to this method include: 1) learners come to class prepared, with the same level of relevant information and knowledge, ready for application [10], 2) student engagement in activities is promoted through active learning sessions, as students spend in-class time applying knowledge and theory previously learnt, and 3) student motivation is increased, as they are accountable to each other for their contribution to activities [10]. Our program was planned to increase student knowledge and skills through pre-class preparation, followed by a face-to-face session, including small group activities, peer collaboration and formative assessment with feedback.

Theories that inform educational practice offer valuable lenses to assist in analysis of teaching and learning methods [11]. The Experience-Based Learning (ExBL) model developed by Dornan and colleagues (2014), suggests that medical student learning outcomes are acquired through participation in authentic activities, where resources are creatively used to construct ideal conditions for learning [12]. According to the ExBL model, students’ learning processes are fostered by three key areas of support:

1. *Organisational;* ensuring that the learning experience sits appropriately within the curriculum, with opportunities to participate in practice.
2. *Pedagogic;* is provided by teachers in the learning environment, including mentors, supervisors, and role models.
3. *Affective;* is provided by a warm and inclusive learning environment.

The purpose of this study was to demonstrate the design and implementation of an interprofessional PTT program, and explore outcomes and participant perceptions, using the ExBL model as a theoretical lens.

## Methods

### Course design

In 2016, at the time of the study there was no systematic preparation of students at our institution in interprofessional learning. An interprofessional team of academics from across three healthcare faculties: Medicine, Pharmacy and Health Sciences developed and implemented a *flipped learning*, *interprofessional* Peer Teacher Training (PTT) program. Designed within an interprofessional context, this unique program provides opportunities for healthcare students to develop skills in teaching, assessment and feedback, in preparation for peer assisted learning activities, and interprofessional practice as graduates. Delivered as a six module program (outlined in Table 1), participants were provided with theoretical background and opportunities for active participation in small group interprofessional learning teams. We focussed on using specific frameworks, such as Pendleton’s model [13] to give and receive feedback; Peyton’s four step approach [14] to teach a clinical skill, and ISBAR (Introduction, Situation, Background, Assessment, Recommendations) to effectively communicate clinical handover [15]. Importantly, formative assessment of teaching skills was woven throughout the program. The learning outcomes of the program are listed in Table 1.

**Table 1:** PTT program outcomes and modules

Inter-professional Peer Teacher Training Program		
*Overall program outcomes:* • Develop the teaching and assessment skills required for health professional students to participate in peer teaching and assessment activities • Develop the skills required of health professional students to provide effective feedback to their peers • Assist health professional students to recognise opportunities for teaching and learning within clinical settings, and contribute to the knowledge and skill development of their peers • Provide and encourage opportunities for inter-professional learning and team collaboration that can be applied to professional practice		
*Module 1:* Introduction to the peer teacher training program and effective feedback This module provides a brief introduction to the Peer Teacher Training program. It also provides the foundation for skill development in the area of effective feedback in the clinical setting.		
Delivered completely online: 2 h	Activities: • Complete a discussion board activity • Introduction to the program • A focus on provision of feedback	Tools: • PowerPoint • Videos (examples of how to provide feedback using Pendleton’s model)
*Module 2:* Planning and delivering a teaching session This module introduces the central concepts of teaching plan development and delivery. Participants are provided with opportunities to develop and practice their teaching skills.		
*Face-to-face* *class* *1.5 h*	Activities: • Large group teaching • Small group activities where each student is required to deliver a delivering a 5 min teaching session using Set, Dialogue, Closure • Formative assessment with feedback	Tools: • PowerPoint • Videos with examples of small group teaching • Formative assessment sheets
*Module 3:* Teaching a skill This module provides opportunities for participants to develop competence in skills teaching. Effective feedback and ways in which to determine competency, and improve skill performance are also explored.		
*Face-to-face* *class* *1.5 h*	Activities: • Large group teaching • Small group activities, delivering a 5 min skills teaching session using Pendleton’s model. • Formative assessment and feedback	Tools: • PowerPoint • Videos (examples of how to teach a skill using Petyon’s method) • Formative assessment sheets
*Module 4:* Assessment and feedback in the clinical setting This module provides participants with the opportunity to develop their understanding and skills in the area of assessment and feedback, particularly within the clinical setting.		
*Face-to-face* *30 min*	Activities: • Large group teaching	Tools: • Powerpoint
*Module 5:* Small group teaching This module emphasises key skills and strategies needed in small group teaching, with an emphasis on inter-professional teaching.		
*Face-to-face* *1 h*	Activities: • Large group teaching	Tools: • Powerpoint • Videos with examples of inter-professional teaching
*Module 6:* Effective clinical handover This module emphasises key skills and strategies needed for effective clinical handover.		
*Face-to-face* *1.5 h*	Activities: • Large group teaching with Powerpoint • Small group activities where each student performs a clinical handover using the ISBAR model. • Feedback	Tools: • Powerpoint • Videos with examples of clinical handovers • Scenarios for role play

#### Facilitators

At each face-to-face session, a total of seven facilitators were present. We had a pool of trained facilitators to draw from for each session. Four academics from medicine, two from pharmacy, and two from allied health were available to lead facilitation. Additionally, four junior medical doctors and three final year medical students who had completed a teacher training program previously were available both to assist with facilitation of small group activities, and develop their own peer teaching skills. All facilitators were provided with a 1 h orientation session, and were provided with all teaching material prior to participation.

#### Mode of delivery

The PTT program used a *flipped* mode of delivery. Students had online access to all course material via the University’s learning management system. This included the content of each of the six modules, activities, and seven videos providing guidance on how to teach a skill; how to provide feedback; and how to teach small groups in the clinical setting. Students were required to complete module 1 online prior to attending a 1 day face- to- face class. The all day face-to-face teaching was a mixture of both small group (*n* = 3–4 students per group), and interactive, large group sessions. In total, we ran four face-to-face classes (*n* = 90).

#### Assessment and feedback

Formative assessments with feedback took place throughout the PTT program. Students were required to complete online tasks during module 1. In class, in their small groups, students presented a pre-prepared 5 min teaching session on a healthcare topic, and a 5 min skills teaching session on a non-healthcare topic (for example, making a paper boat), requiring pre-class preparation of approximately 2 h. These activities were formatively assessed using prepared marking rubrics during small group sessions, and students were provided with immediate feedback from the facilitator. Students were also required to provide formal feedback to their peers during these small group activities, and were formatively assessed on their ability to do so, and provided with constructive feedback. In their small groups, students also practiced clinical handover utilising ISBAR, using clinical scenarios. For example, a scenario where a speech pathologist, after assessing an in-patient to be at risk of aspiration, contacts the medical team to discuss the patient’s management plan.

#### Certificate of completion

At completion of the course, students received a certificate as evidence of formal training in teaching and assessment; plus a hard copy of all course content.

### Study design

#### Recruitment

Senior students from the faculties of medicine, pharmacy and health sciences at the University of Sydney, were invited by email to take part in the PTT program. They were required to register online for the program.

#### Data collection and analysis

##### Pre and post-course questionnaires

Quantitative and qualitative data were collected from student participants by pre and post program questionnaires (see supplementary file to view the questionnaire). Unsurprisingly, there is no available, validated instrument for assessing our PTT program. Consequently, we generated our own questionnaire, based on three key themes we identified when designing the PTT program. The questions for students to reflect on were designed around these three themes, specifically:

1. Participants’ perceived ability with respect to the learning outcomes of each module, such as *“I feel confident to provide constructive feedback to my peers.”*

2) Participants’ intention to take part in future peer tutoring activities, such as *“I am likely to volunteer to take part in formative peer assessment activities”.*
3) Participants’ attitudes towards interprofessional learning were measured using questions from the Readiness for Interprofessional Learning Scale (RIPLS) [16]. For example; *“Shared learning with other healthcare students as a student will help me to become a better team worker”.*

The pre-course questionnaire was delivered online via LimeSurvey, and the post-course questionnaire was completed by students using a paper-survey at the end of the face-to-face class. Both closed and open-ended questions were used. For closed items, we used a five-point Likert scale ranging from ‘strongly disagree’ (1) to ‘strongly agree’ (5). Quantitative data were analysed using descriptive statistics. The software package used was SPSS, version 22.

Participants were asked to respond to open ended questions pertaining to 1) the most useful aspects of the PTT program, 2) suggestions for improvement to the PTT program, 3) the positive and negative aspects of working with other health professional students. A thematic analysis of the qualitative data was performed [17] within each category. Data were coded and categorised into themes by the first and third authors (AB and CvD), and the data within each theme were quantified in order to measure thematic prevalence [17].

### Ethics approval

The University of Sydney Human Research Ethics Committee approved the study. Written consent for participation was obtained from participants to enable us to include their data from this study.

## Results

### Registration and demographics

In total, 115 health professional students registered for the PTT program. Of the 115 students who registered for the PTT program, 90 (78%) successfully completed the PTT program. Thirty eight of these students (42%) were medical students, 34 (38%) were pharmacy students, and 18 (20%) were allied health students (physiotherapy and speech pathology). Thirty three of the students (37%) were male, and 57/90 (63%) were female. The median age was 23 years, and the range was 19–36 years.

### Pre-course and post-course questionnaires

**Fig. 1:**
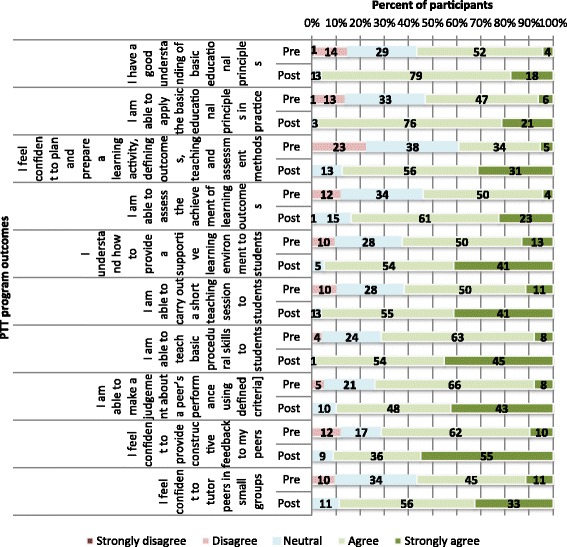
Participants’ pre-course (*N* = 115) and post-course (*N* = 80) perception of Peer Teaching Training (PTT) program outcomes

**Fig. 2:**
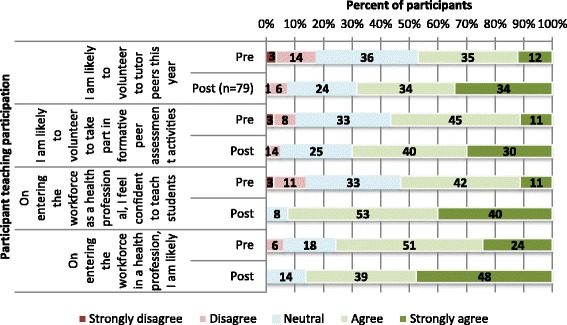
Participants’ pre-course (*N* = 115) and post-course (*N* = 80) students’ intention to participate in peer teaching activities

**Fig. 3:**
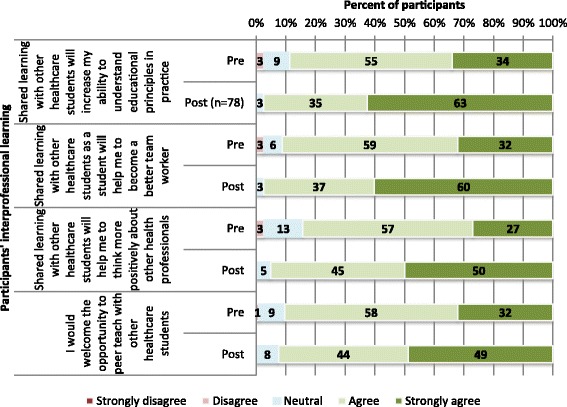
Participants’ pre-course (*N* = 115) and post-course (*N* = 80) perception of their interprofessional learning

Of the 115 students who had registered for the program, all (100%) had completed an anonymous pre-course questionnaire. Of the 90 students who attended and completed the program, 80 (89%) completed the post-course questionnaire. The results of pre-and post-questionnaire are reported in Figs. 1, 2 and 3:

1. *Student experiences of learning outcomes:*

Student responses to questions regarding program outcomes (both pre-and post-course) are provided in Fig. 1. It is clear from the figure students reported vast improvements in their perceived competence and confidence in their understanding of educational theory, ability to carry out a short teaching session, ability to teach basic procedural skills, ability to assess, and ability to provide constructive feedback.

2) *Student intentions to participate in peer teaching and preparedness to practice*

Student responses to questions regarding their intention to participate in peer tutoring activities (both pre- and post-course) are provided in Fig. 2. Again it is clear from the figure that students reported a substantially increased likelihood of their intention to take part in peer tutoring and peer assessment activities at University, and an increased likelihood of intention to teach and assess students on entering the workforce. They also reported an increased confidence to teach students on entering the health professional workforce.

3) *Student attitudes towards interprofessional learning:*

Student responses to questions regarding attitudes towards interprofessional learning (both pre- and post-course) are provided in Fig. 3. It is clear from the figure that following participation in the PTT program, students reported a far more positive attitude towards interprofessional learning, and an increased awareness of interprofessional teamwork requirements.

### Responses to open ended questions

Participant responses to open ended questions are displayed in Tables 2, 3 and 4. Qualitative data is presented within themes in each of these tables, with the data quantified to measure thematic prevalence [17]. Table 2 presents students’ perceived “Most useful aspects of the Peer Teacher Training Program”. In summary, the most useful aspects were perceived to be the models provided for teaching, feedback and communication (37/80, 46%); the small group activities, with provision of a safe environment for practice, self reflection and feedback (47/80, 59%); the content, resources and teaching methods (19/80, 24%); and the Interprofessional aspect of the program (20/80, 25%).

**Table 2:** Students’ perceived “most useful aspects” of the PTT program

Explanation of the theme	Theme and students’ comments	No. of similar responses
	The models provided for teaching, feedback and communication	37/80 (46%)
The use of frameworks in teaching, feedback and communication (Pendleton’s model of feedback; Peyton’s four step approach to teaching a skill; and ISBAR for handover) It was noted that these frameworks would assist in inter-professional health care communication.	*I think the practical skills were great- extremely useful. It helped highlight the effectiveness of using a framework and helped reiterate a structured approach towards teaching and communicating thoughts and ideas.* *ISBAR method of communication allowed me to develop a method by which I can succinctly communicate with other HCPs; especially in a community pharmacy setting where as a pharmacist I need to often talk to busy doctors.* *I enjoyed the use of models as a structure to give feedback and teach a skill. Also, I very much enjoyed ISBAR and think this will be extremely useful in the future. It will help me to interact with other HCP more efficiently.*	
	The small group activities requiring pre-preparation, and multiple opportunities to give and receive feedback	47/80 (59%)
The active small group sessions provided a safe environment to practice skills and reinforce theory. Students found pre-class preparation for the small group activities useful. The practical sessions provided an opportunity to not only practice skills, but also practice giving and receiving feedback Opportunities for self reflection and improvement were provided through multiple feedback	*Small groups provided a good learning environment, it was more comfortable and engaging. Able to apply theory learnt in a practical manner.* *The small group tasks and physical application of newly learnt skills with an educator present firmly grounded these basic principles of teaching in my memory.* *The practical components which followed the framework we were taught to use were very helpful. Getting the opportunity to prepare teaching sessions, provide feedback and do handover was very helpful.* *Reviewing the principles behind creating a teaching session and practicing giving feedback - solidifying positive feedback and giving constructive feedback. Providing good constructive feedback and receiving them provide an opportunity to self reflect on aspects that I think I can improve on.* *Giving us the chance to practice the skills taught to us and be provided with individual feedback was very effective. Opportunities to practice basic teaching principles (*i.e. *set, dialogue, closure and the 4 step approach to skills teaching), and receive feedback for this from both a peer and a facilitator.*	
	Inter-professional aspect of the program	20/80 (25%)
Provision of a formal platform to interact with other health professionals Helped to develop an understanding of inter-professional communication strategies Helped to develop an understanding of the roles of other health professionals	*The opportunity to interact with students outside my discipline throughout the day-during activities and breaks,* to gain perspectives on how other professions approach a scenario of what actions they face. *It helps me to understand the necessary and tricks for interprofessional communication with other healthcare professional (the language, scope and context). The importance of using ISBAR when interacting with other healthcare professional.* *Gain insight about the level of understanding and background knowledge that students from other disciplines. Talk to other health professional gain their insight learn new thing. I found being able to gain an understanding and knowledge of other health care fields very satisfying and it changed my previous perspective about what other health professionals were involved in*	
	The content, resources and teaching methods	19/80 (24%)
The structure of the face-to-face session assisted in student learning The variety of teaching methods, and resources helped to students to prepare and actively participate in class. Students felt the program provided adequate foundation to build on and practice.	*The layout of the whole day- the flow of the information was great to understand what was coming up next. The small group tasks and physical application of newly learnt skills with an educator present firmly grounded these basic principles of teaching in my memory.* *Going through the steps used in teaching. Using different materials, and tasks to go through the steps* e.g. *discussions, video, small group activities. The videos were exceptional and it was great to see examples on how to do things well. Clear instructions from staff. Good delivery of background knowledge. Good powerpoint presentations. Involving students in the teaching* i.e. *questions, group activities. Overall, very good and informative course.* *This program really started with what they expected us to know and slowly built up small goals and achievements throughout the day. Extremely engaging and very useful. Taught many skills that should be applicable in everyday teaching. Overall, extremely beneficial. Thank you so much for today!*

**Table 3:** Suggestions for improvement to the “Peer Teacher Training” program

Explanation of the theme	Theme and students’ comments	No. of similar responses
	Increased small group teaching, and more content delivered online	
Students would like more theory delivered online, to reduce face-to-face teaching time, and increase small group activity time.	*Even less didactic teaching. Have it prepared online, mostly focus on workshop sessions and have a shorter day.* *I think the peer teacher training program can be improved by providing more opportunities for students to showcase the learned skills. Reduce the amount of lectures. Shorter day.*	30/80 (38%)
	Additional health professional students included	
Students would like to see more health disciplines included Additionally, they would like to increase their knowledge of the various health professional curricula Greater mix of students in each small group to mirror hospital activities	*I think having the opportunity to engage with more healthcare professionals and do more small teaching sessions would really help more towards our future profession in term of communication and feedback to help us learn for the next time.* *More discussion into what role each student has and the depth of knowledge expected as I wasn’t sure what year the other students were and what prior knowledge they have.* E.g. *as a pharmacy student, I wasn’t sure how they taught the med students in terms of syllabus.* *I wish there was more allied health professionals in this session as they really helped to create a real environment as it usually is in the hospital.*	16/80 (20%)
	Integrate the PTT program into the health professional curricula	
Some students commented that more students should be given the opportunity to participate, and the PTT program should be part of the curricula across the health professions	*They should integrate such program into the core curriculum for all healthcare discipline. So when they graduate and practice in their respective discipline they can too be better teachers.* *I think it was fantastic, and very important for healthcare students, so maybe if resources are available to take in more students, increase the awareness surrounding the program as it provides a fantastic overview and foundation of teaching skills.*	10 (13%)

**Table 4:** Most positive and difficult aspects of working with other health professional students

Positive		Negative	
Theme: Gaining an understanding of the roles of multi-disciplinary team work and holistic patient No. of similar comments 24/80 (30%)		Theme: No Negative aspects to working with other health professional students No. of similar comments 45/80 (56%)	
The inter-professional nature of the program helped students to gain an understanding of how health care professions will work together in multi-disciplinary teams within health systems in the future This also helped students to gain a holistic view of patient care Understanding the roles of other health professionals, and how to develop teamwork skills	*Understand how other disciplines work together to achieve the same goals. Learning about other health topics not related to your discipline.* *I learnt the different roles of other professionals and it allows me to better understand working in a multi-disciplinary team and what it encompasses* *Working with other health professional students replicates real life hospital settings where multidisciplinary teamwork is used.* *It’s common to learn on paper but never really practiced in real life. I get to know their perspectives toward health care.* *Helps us work together to improve multiple aspects of a patient’s care- holistic care. Learning other professions perspectives on patients’ needs and how to fulfil them.* *I was able to appreciate what my discipline of speech pathology has to offer to other health disciplines (pharmacy and medicine) and how it fits in to the overall spectrum of healthcare*. *Interacting with other sets of people rather than from our own discipline seeing how our role fits in with theirs, and how we aid in their clinical decisions.*	Most students commented that there were no negative aspects to working with health professional students from different disciplines, and identified positive aspects	*Nope. I thought it was on important aspects of this workshop… it was a great experience. Loved listening to the stories from the other HCP students even the lectures.* *No, working with other HCP students helps build good rapport and develops a positive attitude to bring across when practicing in the real healthcare industry.* *Can’t think of any! I think it is absolutely important for students from different health schools to interact (Just like how multi-disciplinary teams work together in hospital). Thank you for the course.*
Theme: Develop inter-professional appropriate communication skills No. of similar comments: 18/80 (23%)		Theme: Differing levels of knowledge and experience No. of similar comments: 13/80 (16%)	
Developing a knowledge of the roles of other health professions; Comparing and developing knowledge and appropriate communication skills when dealing with other health professions Highlighted the importance of communication skills, for example, in handover, and the importance of avoiding jargon	*I like talking to other people and hearing their experience and being able to compare my knowledge/communication skills- very important given we all work together.* *I was able to improve my own communication skills, learn about their courses and how professionals can work together to deliver the best healthcare to the patient.* *Being mindful about our terminology when teaching during teaching a health topic exercise. Sharing our experiences and knowledge.* *Being exposed to other professions equals increased understanding of what they know and the level of communication you should use.* *Working with other professional students made me understand the importance of avoiding professional jargon and to use more general terms in addressing topics so that not only pharmacists, but also other non-pharmacy students could comprehend.*	Different levels of knowledge and experience, making it difficult to ‘pitch’ a teaching session at the right level Some students suggested more time could be given to explaining the roles of other health professions.	*Since this was one of the fist times I had more exposure with other professions, I didn’t know how much they knew.* *Everyone has different backgrounds so some students knew more than others in particular topics. Not necessarily negative, however, presenting a medical-related case to non-medico’s as well as medical students, makes pitching the appropriate level difficult.* *Maybe more time could be allocated to understanding more about the professions and their role in healthcare.*
Theme: Develop an understanding and appreciation of the curricula, knowledge and skills of other disciplines No. of similar comments: 40/80 (50%)			
Learning about the coursework and curriculum other disciplines Gain an understanding of the differences and similarities in clinical knowledge and experience Developing an understanding of how the roles of individual disciplines contribute to holistic patient care Realising that others appreciate each others roles	*Finding out what they learn in their curriculum so that in the future we can communicate to them effectively* e.g. *by not using jargon they don’t understand.* *Sharing different stories and reflections of their respective degrees. Discovering the differing scopes of each discipline. Understanding the teaching provided to other health professional students and their knowledge base and strengths will greatly improve my interactions with them in the future.* *Learning from others has given me a better understanding of health topics outside the scope of medications (*e.g. *speech pathology for stroke recovery,* etc.*). It added to our understanding about other professions.* *I got to understand through their goal when it came to a scenario* e.g. *patient falls and breaks wrist- pharmacy = what medications were they on, was it affecting their brain/consciousness. Physiotherapy = which angle was the fall, which bone was involved.* *Learning about their line of work and an aspect of their course. Being mindful about our terminology when teaching during teaching a health topic exercise. Sharing our experiences and knowledge.* *You have a better insight to how they are trained and the difficulty they may experience when dealing with a particular situation. Also have a good cross-junction dialogue and patient safety.* Their different approaches to small group teaching. The type of clinical expereince and clinical skills they learn. How our professions overlap- more so than I was first aware!		

Table 3 presents students’ “Suggestions for improvement to the Peer Teacher Training Program”. These included more theory to be delivered online to reduce face-to-face teaching time, and increase small group activities (30/80, 38%); the inclusion of more health disciplines, and a more diverse mix of students within small groups (16/80, 20%); and integration of the PTT program into the health professional curricula (10/80, 13%).

Table 4 presents both “The most positive and the most difficult aspects of working with other health professional students”. The most positive factors included gaining an understanding of the roles of multi-disciplinary team work and holistic patient care (24/80, 30%); developing interprofessional communication skills (18/80, 23%); and developing an understanding of various health discipline curricula and training. Over half of the students [45/80 (56%)] commented that there were no negative aspects to working with other health professional students. Difficult aspects included the various levels of knowledge and experience, and the desire for more time devoted to explaining the various roles (19/80, 24%).

## Discussion

This study sought to explore factors in a flipped, interprofessional PTT program that enabled a diverse range of health professional students to simultaneously achieve key learning outcomes in educational theory and teaching skills, while developing and consolidating interprofessional practice, and their preparedness for peer teaching practice. Our findings showed that students were able to achieve the PTT program learning outcomes by demonstrating and having feedback on various aspects of key teaching competencies. Students felt prepared to practice as peer teachers, and many had undergone a change in attitudes toward interprofessional learning. As reflected in our results, students perceived the PTT program to be well aligned with their respective curriculum. The learning environment was enriched by a framework that allowed participants to prepare, practice and improve their newly acquired knowledge and skills in teaching, assessment and feedback within an interprofessional context. While there is some overlap, we now discuss the implications of our findings in the context of the three critical supports for Experience-Based Learning (ExBL): 1) Organisational, 2) Pedagogic, and 3) Affective.

### Organisational support

Organisational support ensures student learning experience is aligned with curriculum outcomes, with opportunities for active participation [12]. A clear strength of the PTT program was its flipped learning format. Students felt the required pre-class preparation, including online pre-reading, discussion board, videos, and online activities enhanced their face-to-face learning experience, and promoted active participation. By using a Learning Management System, order and commitment were gained from students prior to class, as students became familiar with the required activities, assessment methods, and feedback techniques. Students felt the course content was relevant, with an appropriate depth and breadth of theory provided. They appreciated being able to attend a structured, formal class, with receipt of a certificate considered a valuable addition to their resume. Students felt that the program was well aligned with their respective curricula. However, development of teaching skills and provision of interprofessional activities were not otherwise provided at University. In fact, some students indicated a desire for wider opportunities for participation in the program, with 16/80 (20%) suggesting a greater range of healthcare professions be included. Some (10/80, 13%) suggested the program be embedded across healthcare curricula. In order to align with twenty-first century graduate requirements, there is a responsibility to ensure health professional students develop professionalism skills in both teaching and [18] and interprofessional teamwork [19].

### Pedagogic support

Pedagogic support includes support for practice-based learning, which is provided by teachers in the learning environment [12]. Sixty percent of students in our study commented that provision of small group activities enhanced the achievement of their learning outcomes. The interactions of senior students from across the health professions, sharing their experiences, promoted collaborative student engagement. The small group activities required students to teach a skill, teach a medical topic, and provide feedback to peers. Evidence suggests that active learning opportunities that engage students provide a deeper understanding of knowledge, and aid knowledge retention [20, 21]. Our students appreciated the opportunity to model what they had learnt in theory. Students (37/80, 46%) reported the frameworks and models used in the PTT program, such as Pendleton’s model of feedback [13], Peyton’s four-step approach to teaching a skill [14] to be useful tools that will assist them in the future. In particular, they found ISBAR [15] for handover valuable in developing interprofessional communication skills, including the use of specific terminology. The PTT program facilitated students’ understanding of other health professions, helping to develop a more positive view of interprofessional learning. Students reported an increased understanding of how the individual roles of each health profession contribute to the function of multi-disciplinary teams, to provide holistic patient care.

### Affective support

Affective support is provided by a warm and inclusive learning environment [12]. When students feel they are being treated as members of one community, with similar goals, a sense of belonging is fostered [22]. Students from a range of disciplines came to class prepared, with meaningful and manageable tasks that contributed to the learning of others in small group settings. The learning environment afforded by the facilitators promoted supportive and constructive interactions among group members that fostered students’ confidence in their teaching skills. Recent systematic reviews of teacher training programs for health professional students identified lack of participant assessment and feedback as a common deficit [1, 2]. However, in the PTT program, student learning was enhanced through multiple opportunities for practice with immediate feedback. By allowing students to provide and receive feedback, their responsibilities were aligned with their abilities, and competence and confidence was developed [23]. Students became active participants in developing their own skills in the relative “safety” of small groups. However, in addition to initial training, acquired skills require ongoing reinforcement and practice [3, 24]. Although students were provided with at least three small group activities, 30/80 (38%) expressed a desire for more of the educational theory to be delivered online, in order to provide additional opportunities for practice with feedback in small groups. Students’ comments are aligned with the recent trend towards the “flipped classroom” model, suggesting increased student satisfaction, engagement and learning outcomes [10].

## Limitations

The strength of this study is that it is one of the first to demonstrate the feasibility and effectiveness of an interprofessional peer teacher training program. We acknowledge that the students who participated in the PTT program had voluntarily chosen to do so, which may have biased our results. We also acknowledge that our data collection instrument was purpose-built, with its reliability and validity being unknown. For reasons beyond our control, there was a high drop-out rate from those students who had registered (*n* = 115) compared to those who attended (*n* = 90). This large drop-out rate prevented us from carrying out any formal before-after statistical testing. Unfortunately we were unable to distinguish those 35 students who completed the pre-program questionnaire, but did not attend the program, or did not complete a post-program questionnaire, from the other 80 students. In our attempt to maximise student responses, and to gain honest responses from students, our data collection methods and tools had ensured all student responses to questionnaires remained anonymous, making it impossible to identify respondents or to match data. Since we have no information on the 25 students who dropped out between registration (pre-test) and the actual 1 day teaching session, we cannot test whether they differ from those who completed the course. Although it is possible they were systematically different from those who completed the course, we feel this is unlikely. Consequently, for the purposes of comparison we have simply assumed those who dropped out had similar pre-test results as the remainder. Thus, in Figs. 1, 2 and 3 we have used the data from all 115 in the pre-test results, and simply compared their results descriptively with the 80 who completed the course and supplied post-test data. However, we judged it inappropriate to perform formal statistical testing. This large drop-out rate also created difficulties with administration of the program. For example, it proved difficult to plan an equal distribution of students from the various disciplines within small and large groups. Although it would clearly consume the limited available face-to-face teaching time, to avoid this problem in the future, the pre-test should be performed only on those who attend on the day of teaching session. Alternatively, rather than making their responses anonymous, we could choose to de-identify responses.

## Conclusion

The PTT program provided a framework where students could develop and practice their teaching skills, helping to shape students’ professional values as they assume peer teaching responsibilities and move towards healthcare practice, where inter-disciplinary team work is required [25]. Students from across three health faculties demonstrated a common interest in improving their teaching, assessment and feedback skills, and actively participated in the program. The flipped learning, interprofessional format was successful in developing students’ skills, competence and confidence in teaching, assessment, communication and feedback. Importantly, participation increased students’ awareness and understanding of the various roles of health professionals. The PTT program provided a dynamic tool to provide and shape opportunities for interprofessional activities, positively impacting on the culture of the health profession faculties at the University of Sydney. It is now our intention to widen participation and extend opportunities for practice.

## Abbreviations

ExBL: Experience-Based Learning
PTT: Peer Teacher Training
